# Effects of Konjac Glucomannan/ε-Polylysine Hydrochloride/Ferulic Acid Composite Coating on the Freshness Preservation Performance and Flavor of Refrigerated Sea Bass Fillets

**DOI:** 10.3390/foods12030517

**Published:** 2023-01-23

**Authors:** Huibao Xiao, Jun Liao, Yongshi Chen, Xiuping Tong, Xiangyun Sun, Jiqiang Yan, Jie Pang

**Affiliations:** 1 College of Food Science, Fujian Agriculture and Forestry University, Fuzhou 350000, China; 2 College of Computer and Information, Fujian Agriculture and Forestry University, Fuzhou 350000, China

**Keywords:** sea bass, konjac glucomannan, ε-polylysine, ferulic acid, flavor quality

## Abstract

Coating preservation has a remarkable effect on the preservation of aquatic products. This work prepared a composite coating using konjac glucomannan (KGM) as the film-forming matrix and ε-polylysine hydrochloride (ε-PL) and ferulic acid (FA) as the preservative. Three types of treated sea bass (KGM, KGM-ε-PL, and KGM-ε-PL-FA) and untreated sea bass were stored at 4 °C for 20 days to compare freshness changes under different treatment conditions. The results showed that the surface color and texture of sea bass in refrigerated storage changed dramatically and deteriorated as storage time increased. The composite coating treatment was significantly different from the control group. Using Gas-phase ion migration spectrometry (GC-IMS) technology, 32 volatile compounds, such as aldehydes, alcohols, and ketones, were found in fillets during flavor quality analysis. The composite coating can successfully inhibit the formation of odor compounds such as 2-nonenone, isoamyl alcohol monomer, ammonia, and trimethylamine, delaying the deterioration of fish and improving freshness. Among them, KGM-ε-PL-FA composite coating has the most remarkable preservation performance, which significantly inhibits the occurrence of rotten odor, and has a potential application prospect in the field of food preservation.

## 1. Introduction

The Chinese sea bass (Lateolabrax maculatus) is mainly from the Asian, yellow, and Bohai sea areas [1]. It is a major marine fish farmed in China’s coastal areas. Its meat is tender and delicious, and high in protein, vitamins, unsaturated fatty acids, and minerals. It has a high biological and nutritional value [2]. However, since fish contain biological macromolecules such as proteins and fats, decomposition reactions occur during storage and transportation after the fish die. The production of ammonia, ketones, alcohols, aldehydes, and other substances directly affects seabass meat's sensory and flavor quality [3]. Many studies have shown that composite coating has a pronounced effect on the preservation of aquatic products because the layer has the advantages of effectiveness, low cost, and safety [4,5,6]. Most studies use fundamental physical and chemical indexes as evaluation criteria, such as pH value, Total volatile base nitrogen (TVB-N) value, Thiobarbituric acid reactants (TBARS) value, and other traditional indexes. However, there are few reports about the effect of KGM coating on preserving the flavor and quality of sea bass fillets. Currently, in the study of volatile organic compounds in seafood, the most advanced instruments are used to analyze and evaluate the flavor quality of seafood products. KGM is a natural polysaccharide safe for living things and makes great films. The topic of KGM coating carriers has received much attention in recent years. However, the physical and chemical properties and antibacterial properties of pure KGM film are poor, which limits its application in the field of food preservation [7,8].

ε-PL is a natural food preservative that is stable in acidic and alkaline environments. Several countries have approved it as a food preservative because it kills bacteria, is safe, and works well with living things [9]. Cai et al. discovered that ε-PL and sodium alginate compound preservatives effectively preserve Japanese refrigerated perch's flesh color and tissue hardness while inhibiting micro-organism growth [10]. Liu et al. studied the effects of ε-PL on the bacterial community and sensory and chemical properties of Bighead carp fillets under cold storage. They found that ε-PL can improve the bacteriostatic effect and delay chemical changes in fish [11]. At the same time, ε-PL has a good effect on preserving other foods, such as making fresh lettuce less likely to leak water [12] and stopping microbes from growing in beef and chicken [13]. FA is a phenolic acid from grain, seeds, leaves, and extracted plant cell walls. It has antioxidant, antibacterial, and anti-inflammatory effects and has been used in the food industry as a preservative [14]. Nicolau-Lapena et al. found that adding FA to the coating film can effectively prolong the quality of freshly cut apples and inhibit microbial growth [15].

This study used KGM composite coating as a film-forming substrate, adding a certain amount of epsilon ε-PL and the FA to form a composite membrane. The preservation effect of KGM composite coating on sea bass is studied through sensory evaluation, color difference determination, texture determination, and volatile component determination. The GC-IMS technology in a qualitative and quantitative analysis, and principal component analysis model is set up, to compare the differences between different samples. This study developed a new type of preservation film with a natural macromolecular material as a film-forming substrate and natural active material. This provides a new idea and scientific basis for preserving aquatic products.

## 2. Materials and Methods

### 2.1. Materials

Konjaku flour was purchased from Yunnan Sanai konjac development company (Zhaotong, Yunnan, China). ε-Polylysine hydrochloride (Mv < 5000), ferulic acid (99.0%), and the 1,1-diphenyl-2-picrylhydrazyl (DPPH) powder (96%) were purchased from McLean Biochemical Technology Company (Shanghai, China). Potassium bromide (AR) was purchased from Sinopharm Pharmaceutical Chemical Reagent Company (Shanghai, China). LB agar was purchased from Qingdao Hi-tech Park Haibo Biotechnology Company (Qingdao, China). Glycerol (AR) was purchased from Guoyao Chemical Reagent Company (Shanghai, China).

### 2.2. Preparation of Composite Coating

100 mL of ultrapure water was placed in a mechanical stirring machine at a stirring speed of 450 r/min. Then, 0.6% (*m*/*v*) KGM was slowly added to pure water and stirred at 50 °C for 2 h to fully dissolve KGM and form a uniform, viscous, transparent solution. KGM coating liquid was prepared by adding 0.2% (*v*/*v*) glycerol to the transparent solution and stirring for 0.5 h. KP composite coating liquid was prepared by adding 0.14% (*m*/*v*) ε-PL to the transparent solution for 1 h and then adding 0.2% (*v*/*v*) glycerol for 0.5 h. The composite coating solution of KPF50 was prepared by adding 0.14% (*m*/*v*) ε-PL and 0.050% (*m*/*v*) FA to the transparent solution and stirring for 1 h, and then adding 0.2% (*v*/*v*) glycerol for 0.5 h. The above three kinds of coating liquid were put in a drying oven at 45 ℃ and dried for 15 h until the film is completely dry.

### 2.3. Treatment of Sea Bass Fillets

We remove the sea bass’s head, offal, and skin, cut both sides of the back ridge, rinsed with water, and dried the surface water of the fish with sterile filter paper. The fish was soaked in a 0.6% pure KGM coating solution for 15 min and then taken out a KGM-treated sample. The fish was bathed in a composite coating solution of 0.6% KGM and 0.14% ε-PL for 15 min and then taken out a KP-treated sample. The fish was immersed in a composite coating solution of 0.6% KGM, 0.14% ε-PL, and 0.050% FA, and the fish was wholly immersed for 15 min and then removed, which has KPF50-treated samples. The fillets were soaked in sterile water for 15 min and removed as controls. The processed fish were individually packaged, labeled, and stored in a constant temperature refrigerator (4 °C), and their quality parameters were assessed at 0, 4, 8, 12, 16, and 20 days, respectively.

### 2.4. Sensory Evaluation

Nine students who had studied the sensory evaluation were selected as judges to score the appearance (X1), smell (X2), and texture (X3) of sea bass. Before sensory evaluation, the judges washed their hands and maintained oral hygiene. Each sample was evaluated once, and each score was denoted as Xi (i = 1, 2, 3). The total score was X = 0.2 × 1 + 0.3 × 2 + 0.5 × 3, and the final score was the average sum of each X. Higher scores indicate fresher fish, and lower scores indicate poorer fish quality. The following table (Table 1) lists the specific scoring criteria.

### 2.5. Color Difference Measurement

Before measuring the surface of fish using a Chroma Meter CR-400, the device must be calibrated. When measuring, the hole of the chromatic aberration meter must be aligned with the surface of the fish, and five measuring sites must be chosen. The color should be characterized using L* (brightness), a* (red), and b* (yellow), with the final result being an average.

### 2.6. Texture Determination

The hardness, elasticity, chewiness, and toughness of fish meat were measured using a P/30 R probe on a square chunk of fish meat 1.5 cm wide. TPA(TA-XT-PLUS) mode, descent speed, test speed, and return speed are 3.0 mm/s, 0.5 mm/s, and 3.0 mm/s, respectively. The test distance is 5 mm, and the trigger force is 5 g.

### 2.7. Determination of Volatile Components

Pretreatment: 3 g fish meat was placed in a 20 mL headspace flask, incubated at 70 °C for 20 min, then heated continuously to 85 °C, 500 μL injection volume. Gas chromatographic analysis conditions: chromatographic column to maintain a 60 °C environment running for 25 min; carrier gas (N2, purity ≥ 99.999%); the chromatographic column was maintained at a flow rate of 2.0 mL/min for 2 min and gradually increased to 150 mL/min. Ion mobility spectrometry conditions: drift gas (N2, purity ≥ 99.999%), flow rate 150 mL/min, IMS detector temperature: 45 °C.

### 2.8. Statistical Analysis

All experiments were repeated three times. Results were expressed as mean ± standard deviation (SD). Origin 2021 software (Stat-Ease Inc., Minneapolis, MN, USA) and SPSS 27.0 software (IBM, Chicago, IL, USA) were used for univariate analysis of variance and Duncan multiple comparisons (*p* < 0.05) for data processing and significance analysis. The volatile components in the samples have been adopted in Laboratory Analytical laboratories Viewer) software and Library Search software for collection and analysis. SPSS 27.0 software was used for the principal component analysis of GC-MS data.

## 3. Results and Analysis

### 3.1. Effect of Composite Coating on the Sensory Score of Sea Bass Fillets

**Figure 1:**
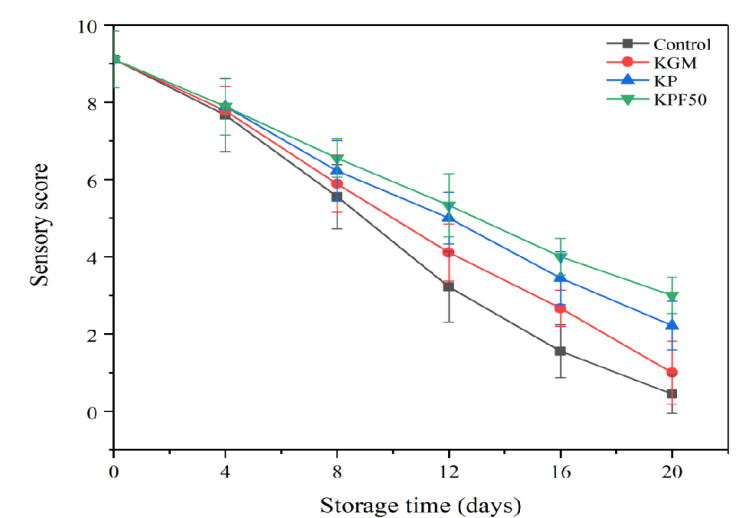
The sensory scores of sea bass fillets during refrigeration storage.

Sensory evaluation can well reflect changes in fish quality during storage [16]. The meat quality of fresh sea bass is bright white, elastic, and has a pleasant aroma. With the extension of time, the fish will deteriorate and produce a strong odor of behavior and rot, which affects people’s purchase intention [17]. As shown in Figure 1, sensory scores of sea bass fillets decreased over time in all treatment groups. On day 4, there was no significant difference among all groups. The sensory scores of the blank treatment group rapidly reduced after the eighth day, and the fish sensory scores of the KPF50 treatment group were the highest. At the first 12 days of storage, the sensory scores of sea bass fillets between the KP treatment group and the KPF50 treatment group were not significantly different. On the 16th day, however, the sensory scores for KPF50 were higher than those for the other treatment groups. It showed that the KPF50 composite coating liquid had more potent antioxidant and antibacterial properties, which could slow the fish’s deterioration. After coating the sea bass fillet with KPPF50 coating liquid, it has no adverse effect on the sensory quality of the fish. It can effectively extend its shelf life for 4–8 days, which consumers can still accept during the shelf life [18].

### 3.2. Effect of Composite Coating on the Color of Sea Bass Fillets

The most direct indicator of fish color and freshness criteria is surface color [15]. As shown in Table 2, At the early stage of storage, the L* value of sea bass fillets in the three coated groups was higher, which may be due to the enhancement of flesh-color brightness by coating liquid on the surface of the fish [19]. In the first four days, all groups had no significant difference (*p* > 0.05). Subsequently, the L* value decreased with the extension of time, and the fish became dark and dull. The changing trend of the a* value is the same as that of the L* value. With the extension of time, the a* value keeps declining, and the fish color is slightly green, indicating that the fish has undergone severe spoilage. The reason may be that adding ε-PL and FA to the KGM coating made it stronger between molecules and made it more compact, which is better for keeping fish fillets fresh [20]. The b* value showed an increasing trend over time. At the end of storage, the b* value of sea bass fillets in the blank processing group was the greatest. In contrast, the KPF50 treatment group had a sluggish upward trend that was substantially different from other treatments (*p* < 0.05). This demonstrates that the coating solution containing the three chemicals inhibits microbial growth and reproduction and efficiently preserves sea bass fillets. In addition, at a later stage of storage, the fish will release a dark yellow cloudy liquid, causing a* and L* values to drop and b* to rise.

### 3.3. Effect of Composite Coating on the Texture of Sea Bass Fillets

**Figure 2:**
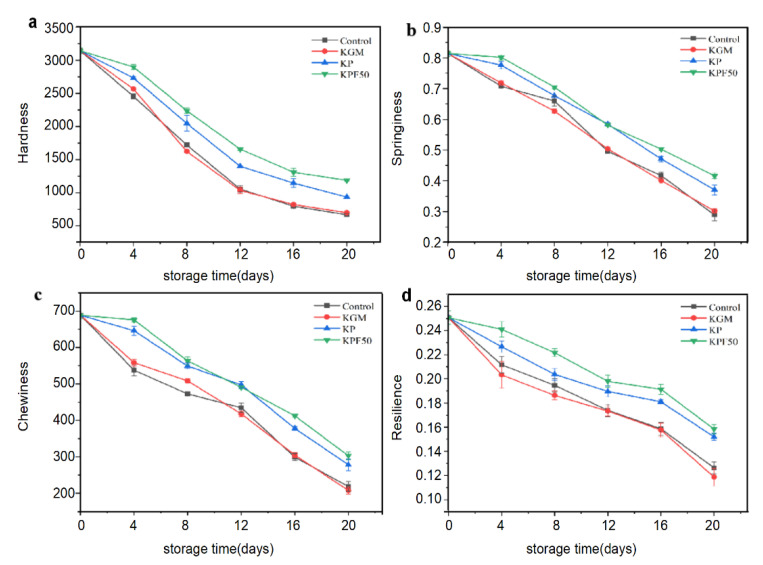
The texture attributes of sea bass fillets during refrigeration storage: (**a**) hardness; (**b**) elasticity; (**c**) chewiness; (**d**) recovery.

Changes in the structure of fresh fish after storage are one of the most critical indicators of freshness [21]. As shown in Figure 2, the hardness, elasticity, chewability, and resilience of the sea bass fillet decreased with the storage extension. It is due to the action of endogenous enzymes and microbes on proteins in fish meat, which disrupt the network structure, resulting in muscle softening and juice loss. As shown in Figure 2a, the hardness of sea bass slices decreased with the extension of time. The fillet hardness of the blank treatment group and pure KGM coating group had no significant difference. It was consistently lower than the KP treatment group and the KPF50 treatment group. At the end of storage, fish fillets treated with KPF50 had higher hardness, indicating that the composite coating inhibited the decomposition of endogenous enzymes and better maintained the hardness of the fish [22]. This is because a thick protective film prevents the exchange of fish and outer material, limiting fish growth and reproduction, slowing the rate of fish glycogen degradation [23], and lowering the hardness of fish. As shown in Figure 2b, the flexibility of sea bass is dramatically reduced with storage time. Compared to other treatments, sea bass slices treated with KPF50 experienced the slightest reduction in elasticity, showing that the composite coating significantly inhibited microbial development. As seen in Figure 2c, the chewiness of sea bass decreased with storage time. Fish fillets in groups KP and KPF50 were chewy. The investigation revealed that the combination of ε-PL and FA might effectively inhibit microbial metabolism and lead to the degradation of protein structure, thereby reducing the degree of spoilage in sea bass fillets. The robustness of fish fillets was dramatically diminished following blank and KGM processing, as seen in Figure 2d. However, the strength of sea bass fillets treated with KP and KPF50 was less than that of the other two treatment groups, thus preserving the meat's textural quality.

### 3.4. Effect of Composite Coating on Volatile Components of Sea Bass Fillets

**Figure 3:**
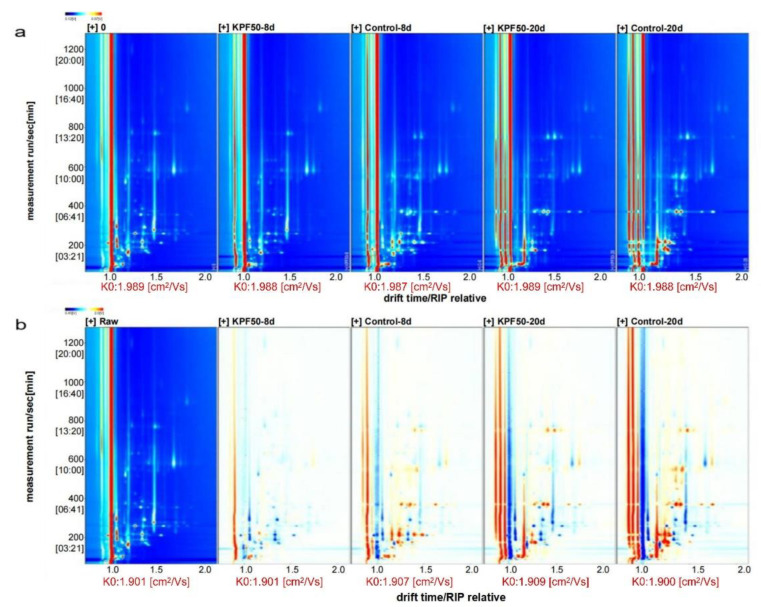
2D GC-IMS spectra of volatile organic compounds in sea bass samples: (**a**) direct comparison; (**b**) difference comparison.

GC-IMS was used to determine and identify volatile components in refrigerated sea bass fillets. The GC-IMS analysis chart in Figure 3 depicts the characteristic flavor of sea bass slices in the blank processing group and the coating treatment group at various storage times. On the horizontal axis of 1.0, the red vertical line represents the reaction ion peak (RIP peak), a dot represents the volatile substance on the right, and the color and area region represent the substance’s content. White represents a lower intensity, weaker ion flow signal, and lower concentration of the essence, while red is the opposite [24,25]. Figure 3b uses the difference comparison model to provide a more intuitive view of group differences. Most of the signals appeared within the retention time of 100–800 s, and new alerts appeared after eight days of storage. The main difference between the KPF50 and new groups was a decrease in the content of characteristic flavor substances. Fish meat, especially unprocessed fish fillets, will deteriorate after 20 days of storage. The degree of spoilage of fish fillets is higher due to their high content of spoilage-like flavor substances.

**Figure 4:**
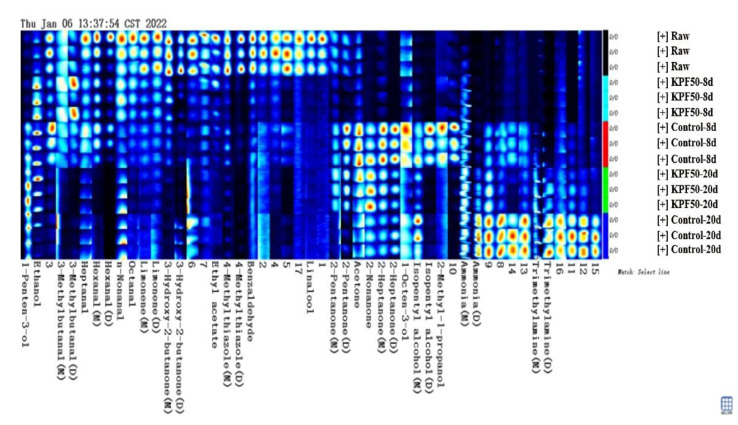
Characteristic peak gallery report of different types of vegetable oil.

To further compare the volatile flavor compounds present in the fish after different storage periods, all peaks in the 2D GC-IMS map were analyzed to establish a fingerprint map (Figure 4). As can be seen from the figure, the brightness of points in the fingerprint represents the change in substance concentration, indicating that there are significant differences in the composition of volatile substances in sea bass fillets with the extension of storage during the refrigeration process. As can be seen from the figure, the flammable substances in fresh sea bass mainly include aldehydes, alcohols, and ketones. At the later stage of storage, the decomposition of these volatile substances continued to decline, and the contents of 3-hydroxy-2-butylone, limonene, octanal, and nonanal in the control group decreased faster. At the later storage stage, the contents of trimethylamine and ammonia with rotten odor increased rapidly. The contents of trimethylamine and ammonia in the coating group were lower than those in the blank group, indicating that the composite coating could effectively inhibit the growth of micro-organisms and the activity of endogenous enzymes in the sea bass slices [26], and improve the preservation effect to some extent.

As indicated in Table 3, 32 compounds, including aldehydes (8), alcohols (7), ketones (8), ammonia (4), hydrocarbons (2), esters (1), and other categories, were found in sea bass fillets at varying storage times (2). In the initial stages of storage, 17 chemicals were discovered. Aldehydes and alcohols were the most abundant substances, indicating that these two volatile compounds were the characteristic flavors of fresh sea bass. Among them, the highest concentrations were octanal, linalool, 3-hydroxy-2-butanone, ethyl acetate, 4-methyl thiazole, and limonene. During the cold storage of sea bass, new peak areas emerged, but several early-stage peak areas faded or diminished. At the end of the storage period, 24 substances were discovered. The peak area of certain chemicals, such as 3-methylbutanal, 2-pentanone, acetone, 2-heptanone, isoamyl alcohol dimer, 1-pentan-3-ol, and 2-methyl-1-propanol, increased and subsequently reduced when storage time was extended. Many compounds, such as 2-nonunion iso-amyl alcohol monomer, ammonia, and trimethylamine, are detected in the middle or late storage. When the concentration of 2-nonanone, isoamyl alcohol monomer, trimethylamine, ammonia, and other chemicals exceeds a particular threshold in fish, an offensive stench is produced [27]. For instance, 2-nonanone and isoamyl alcohol impart the Hara flavor and terrible taste to sea bass meat [28]. Compounds containing nitrogen, including trimethylamine and ammonia, are responsible for the odor of sea bass. After storage, the above substances of sea bass meat treated with KPF50 coating were lower than those of the blank treatment group, indicating that the composite coating inhibited the production of volatile off-flavor compounds of sea bass meat during cold storage.

**Figure 5:**
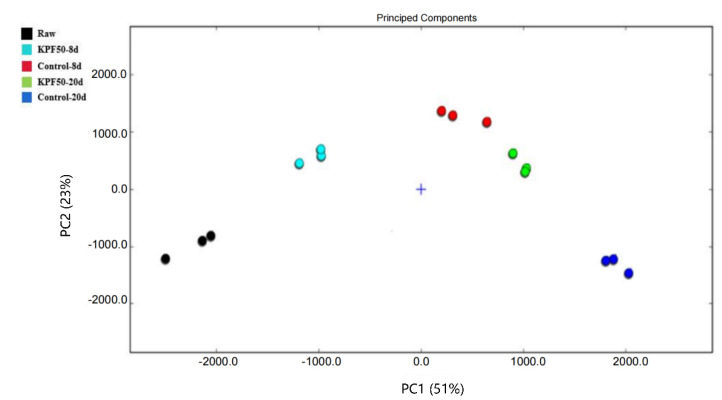
PCA analysis of sea bass fillets samples.

Principle component analysis is building an unsupervised PCA model that represents complex variables by identifying the principal components of some samples and analyzing the rules and differences between pieces based on the contribution rate of the main features. This method generates principal components that are linear combinations of the input variables, effectively reducing the number of variables and removing abnormal data [29]. Figure 5 depicts the construction of principal component analysis (PCA) based on the signal intensity of all samples to further explain the distinctions between volatile components. This graph only displays the classification results for the first two principal components produced by PCA for the example. The 74% contribution rate of PC1 and PC2 thereby indicates that it was facile to distinguish between the sea bass meat samples from different storage periods. In Figure 3, Figure 4 and Figure 5, the distance between samples graphically represents the variations between distinct samples. The near distance between samples suggests a modest difference, while the far distance shows a significant difference. It can be seen from Figure 5 that with the extension of storage time, the volatile organic compounds in sea bass have changed significantly. The volatile components of sea bass treated with coating are closer to those of the blank treatment group, indicating that the composite coating can effectively delay the deterioration of sea bass and extend the shelf life.

## 4. Conclusions

In this study, GC-IMS technology was used to analyze the volatile components of sea bass meat and their changes during storage, as well as to investigate the preservation effect of a composite coating prepared by adding a specific amount of ε-polylysine hydrochloride (ε-PL) and ferulic acid (FA) preservative to sea bass meat with konjac glucomannan (KGM) as the film-forming matrix. Results show that the composite coating has an excellent preservation effect on sea bass, and ε-PL and FA had a synergistic effect, which could significantly improve the preservation effect of KGM film and prolong the shelf life of sea bass meat. 32 Volatile chemicals, including aldehydes (8), alcohols (7), ketones (8), ammonia (4), hydrocarbons (2), and esters, were detected (1). Aldehydes and alcohols are the primary flavoring components in fresh fish meat. Aldehydes and alcohol in sea bass meat in the blank processing group decreased after refrigeration, but ketones and amines increased. Compared to the blank processing group, the KP50 composite coating strongly reduced the development of 2-nonanone, isoamyl alcohol monomer, ammonia, trimethylamine, and other odor compounds, as well as oil and other odors. It demonstrates that the composite film can successfully limit the endogenous enzyme activity, fat oxidation reaction, and microbial growth of sea bass meat, altering its volatile components, decreasing odor, and preserving the original quality of sea bass fillets. The study showed that the composite coating protected the flavor characteristics of sea bass meat.

## Figures and Tables

**Table 1:** Sensory scoring of sea bass fillets.

Quality Parameters	Score		
	10–8	7–4	3–0
Appearance (0.2)	Bright color, full of luster	Dim color, slightly shiny	Dark color, dull surface
Smell (0.3)	Strong umami	Light smell, slightly peculiar smell	Strong stink
Texture (0.5)	Muscles elastic, dense, clear texture	Muscles are more elastic, less dense but not loose	Muscle tissue inelastic, not dense, loose

**Table 2:** Changes in chromatic aberration of sea bass during refrigeration storage.

	Sample	0 d	4 d	8 d	12 d	16 d	20 d
L*	Control	64.42 ± 0.49 ^aA^	63.66 ± 0.85 ^aB^	61.21 ± 0.82 ^bC^	58.91 ± 0.19 ^bC^	56.02 ± 0.19 ^cC^	53.66 ± 0.57 ^dC^
	KGM	64.42 ± 0.49 ^aA^	63.37 ± 0.70 ^aB^	61.9 ± 10.32 ^bBC^	59.25 ± 0.76 ^cBC^	55.05 ± 0.64 ^dD^	52.93 ± 0.71 ^eC^
	KP	64.42 ± 0.49 ^aA^	64.92 ± 0.66 ^aAB^	62.25 ± 0.70 ^bAB^	60.82 ± 0.60 ^cB^	57.75 ± 0.41 ^dB^	55.08 ± 0.16 ^eB^
	KPF50	64.42 ± 0.49 ^bA^	65.51 ± 0.58 ^aA^	63.68 ± 0.59 ^cA^	61.83 ± 0.47 ^dA^	59.08 ± 0.17 ^eA^	56.72 ± 0.40 ^fA^
a*	Control	0.65 ± 0.03 ^aA^	0.43 ± 0.05 ^bB^	0.38 ± 0.02 ^bC^	0.19 ± 0.02 ^cBC^	0.03 ± 0.08 ^dB^	−0.21 ± 0.03 ^eB^
	KGM	0.65 ± 0.03 ^aA^	0.46 ± 0.04 ^bB^	0.39 ± 0.02 ^bC^	0.13 ± 0.03 ^cC^	−0.08 ± 0.09 ^dAB^	−0.24 ± 0.08 ^eB^
	KP	0.65 ± 0.03 ^aA^	0.52 ± 0.02 ^bA^	0.45 ± 0.03 ^bB^	0.23 ± 0.05 ^cB^	0.08 ± 0.03 ^dA^	−0.07 ± 0.11 ^eAB^
	KPF50	0.65 ± 0.03 ^aA^	0.55 ± 0.02 ^bA^	0.51 ± 0.02 ^bA^	0.32 ± 0.05 ^cA^	0.14 ± 0.05 ^dA^	0.33 ± 0.07 ^eA^
b*	Control	−0.49 ± 0.02 ^fA^	−0.21 ± 0.02 ^eA^	−0.1 ± 0.06 ^dA^	0.01 ± 0.05 ^cA^	0.15 ± 0.04 ^bA^	0.30 ± 0.04 ^aA^
	KGM	−0.49 ± 0.02 ^fA^	−0.24 ± 0.04 ^eA^	−0.07 ± 0.02 ^dA^	0.05 ± 0.04 ^cA^	0.17 ± 0.04 ^bA^	0.32 ± 0.05 ^aA^
	KP	−0.49 ± 0.02 ^fA^	−0.33 ± 0.04 ^eB^	−0.23 ± 0.03 ^dB^	−0.11 ± 0.02 ^cB^	0.04 ± 0.05 ^bB^	0.19 ± 0.02 ^aB^
	KPF50	−0.49 ± 0.02 ^eA^	−0.39 ± 0.03 ^dC^	−0.29 ± 0.02 ^cB^	−0.17 ± 0.04 ^bB^	−0.09 ± 0.06 ^bB^	0.14 ± 0.06 ^aB^

Note: different capital letters in the same column represent significant difference between groups at the same time (*p* < 0.05); different lowercase letters in the same line represent significant difference in different time groups (*p* < 0.05).

**Table 3:** Volatile ingredients of sea bass during different refrigeration storage time.

Category	Name of Compound	Molecular Formula	Retention Index	Retention Time/s	Travel Time/ms	Peak Area				
						Raw	KPF50—8 d	Control—8 d	KPF50—20 d	Control—20 d
Aldehydes	nonanal	C_9_H_18_O	1105.00	774.35	1.47	358.16	318.64	191.00	229.85	253.49
	caprylic aldehyde	C_8_H_16_O	1008.30	581.51	1.40	268.00	162.07	103.94	54.94	71.47
	benzaldehyde	C_7_H_6_O	977.50	521.54	1.15	829.05	343.45	384.80	471.61	481.45
	heptanal	C7H14O	900.70	387.24	1.33	201.95	154.55	161.50	94.06	108.94
	Hexanal monomer	C_6_H_12_O	790.20	261.50	1.26	1233.83	810.41	937.81	128.73	290.11
	Hexanal dimer	C_6_H_12_O	789.80	261.13	1.57	624.64	270.46	442.75	38.48	27.60
	3-methylbutylaldehyde	C_5_H_10_O	647.60	163.60	1.17	278.67	532.50	283.53	43.13	79.14
	3-methylbutyraldehyde dimer	C_5_H_10_O	647.60	163.60	1.40	6885.45	8586.27	18,518.25	44,054.62	44,637.79
	1-Pentan-3-ol	C_5_H_10_O	682.80	179.07	0.95	374.45	358.44	1458.22	1912.24	1600.50
	ethyl alcohol	C_2_H_6_O	440.70	96.23	1.05	1106.71	2668.58	1865.12	1811.71	799.76
	linalool	C_10_H_18_O	1105.60	775.77	1.23	115.46	87.26	197.13	133.51	162.63
Alcohols	1-octen-3-ol	C_8_H_16_O	994.80	557.86	1.17	745.18	654.59	465.54	296.75	485.14
	2-Methyl-1-propanol	C_4_H_10_O	618.50	151.83	1.36	446.34	182.43	196.80	211.66	205.66
	Isoamyl alcohol monomer	C_5_H_12_O	737.60	216.67	1.23	65.74	56.01	971.36	65.46	173.03
	Isoamyl alcohol dimer	C_5_H_12_O	734.70	214.39	1.50	14.18	16.07	55.18	21.23	26.32
	2-Methyl-1-propanol	C_4_H_10_O	618.50	151.83	1.36	446.34	182.43	196.80	211.66	205.66
Ketone	3-hydroxy-2-butanone monomer	C_4_H_8_O_2_	742.70	220.68	1.06	3126.38	1537.63	949.28	294.66	360.76
	3-hydroxy-2-butanone dimer	C_4_H_8_O_2_	740.90	219.23	1.33	1594.56	387.69	405.27	189.13	784.18
	2-pentanone monomer	C_5_H_10_O	674.50	175.29	1.12	548.24	242.98	561.07	794.15	404.77
	2-pentanone dimer	C_5_H_10_O	675.10	175.58	1.37	493.73	140.50	930.19	676.32	235.54
	acetone	C_3_H_6_O	470.10	103.79	1.12	1198.01	883.27	3419.88	2928.52	3006.84
	2-nonanone	C_9_H_18_O	1095.50	752.94	1.41	78.20	84.19	492.57	586.55	363.25
	2-heptanone monomer	C_7_H_14_O	891.10	373.15	1.26	32.89	27.97	532.21	296.55	336.05
	2-heptanone dimer	C_7_H_14_O	890.80	372.74	1.63	293.49	403.06	1875.56	1078.46	2163.75
	Ammonia monomer	NH_3_	780.00	252.21	0.89	13,733.51	38,369.74	50,283.61	64,843.40	62,940.28
Ammonia	Ammonia dimer	NH_3_	781.00	253.09	0.85	2453.99	3979.86	9303.66	26,329.55	61,693.84
	Trimethylamine monomer	C_3_H_9_N	519.80	117.90	0.96	295.66	284.48	799.37	4278.75	9233.84
	Trimethylamine dimer	C_3_H_9_N	483.40	107.39	1.15	134.21	169.61	761.16	592.38	392.69
	Limonene monomer	C_10_H_16_	1037.5	633.95	1.2276	143.54	94.92	70.08	57.52	71.03
Alkanes	Limonene dimer	C_10_H_16_	1035.5	630.36	1.29389	199.76	97.39	112.17	87.24	70.15
Esters	ethyl acetate	C_4_H_8_O_2_	590.40	141.28	1.10	399.01	232.80	145.29	83.30	96.60
Else	4-Methylthiazole monomer	C_4_H_5_NS	825.60	296.29	1.06	2888.51	775.18	899.53	380.03	437.78
	4-Methylthiazole dimer	C_4_H_5_NS	823.00	293.58	1.36	1147.53	171.03	235.98	93.57	114.17

## Data Availability

The data are available from the corresponding author.
